# The moderating effect of physical activity on the relationship between neutrophil count and depressive symptoms

**DOI:** 10.1038/s41598-024-63432-x

**Published:** 2024-06-02

**Authors:** Zhaohui Guo, Zhenwen Xie, Peng Wang, Shufan Li, Xin Xin, Xing Wang

**Affiliations:** Shanghai University of Sport, Shanghai, 200438 China

**Keywords:** Physical activity, Neutrophil, Depressive Symptoms, Moderation model, Cell biology, Immunology, Psychology, Diseases, Medical research

## Abstract

Variations in immune cell counts can trigger depressive symptoms, while physical activity effectively reduces the risk and severity of depressive symptoms. This study, based on the NHANES database, analyzes the relationship between neutrophil count and depressive symptoms and explores the moderating effect of physical activity on this relationship. Cross-sectional data from the NHANES database were extracted, including immune cell counts, PHQ-9 scores for self-assessment of depressive symptoms, and Global Physical Activity Questionnaire (GPAQ) scores (PA). The interrelations among physical activity, neutrophil count, and depressive symptoms were analyzed. After controlling for confounding factors, neutrophil count was found to have a significant role in identifying depressive symptoms with an odds ratio (OR) [95% Confidence Interval (CI)] = 1.13 [1.02, 1.251]; the moderating effect of physical activity on the impact of neutrophil count on depressive symptoms was statistically significant (coefficient = -0.0028, *P* < 0.05). Neutrophil count may be a significant factor in identifying depressive symptoms in adults. As an effective moderating factor, physical activity can mitigate the impact of neutrophil count on depressive symptoms to a certain extent.

Depression has become the most significant challenge in global mental health, accounting for 10.3% of the overall disease burden^1^. According to statistics from the World Health Organization, the total number of individuals suffering from depression worldwide has surpassed 350 million^2,3^, marking a 49.86% increase compared to the past. The research indicates that depressive symptoms are considered one of the significant factors leading to the onset of depression. Both physiological and psychological aspects contribute to the body's transition from health to sub-health to disease, ultimately resulting in the development of depression^4,5^. Prolonged lack of effective treatment continuously affects the patient's physical health and quality of life ^6^, while also imposing substantial stress on healthcare resources. Given this context, how to prevent the occurrence of depression and reduce its severity has become an urgent issue for society to address.

The induction of depressive symptoms is influenced not only by factors such as family environment, education level, and marital status^7–9^. Increasingly, studies^10–12^ have identified that inflammatory responses play a critical role in the pathogenesis of depression. Patients exhibit significant specificity in inflammatory markers, metabolic markers, and oxidative stress in their blood compared to healthy individuals^13,14^. This specificity includes changes in the leukocyte subpopulations within the blood immune cells of individuals with depressive symptoms. This occurs because leukocytes, capable of producing large amounts of reactive oxygen species, can generate oxidative stress to eliminate pathogens. Excessive oxidative stress, however, can damage normal cells, including neurons in the brain. If a patient has an elevated white blood cell count, it may lead to increased levels of oxidative stress in the brain, thereby impairing neuronal function and potentially exacerbating depressive symptoms. Furthermore, previous research has found^15,16^ a positive correlation between the severity of depressive symptoms and peripheral blood lymphocytes, leukocytes, and myeloid cells. Meta-analytic results^17^ suggest that an increased relative percentage or absolute count of neutrophils can aid in identifying patients with inflammation-related depressive symptoms. Thus, it is established that immune cell counts can be utilized to identify inflammation-related depressive subgroups to assess the risk of depressive symptoms.

Physical activity (PA) is a mode of action that achieves health promotion, physical fitness enhancement, and improved quality of life through various physical behaviors^18^. Research has found that PA not only significantly influences physiological mechanisms but also enhances positive emotional states and markedly improves depressive symptoms^19^. Furthermore, studies show that moderate physical activity can effectively reduce the risk of depression. This reduction is attributed to the increased expression of adult hippocampal neurons and neurotrophic factors, such as brain-derived neurotrophic factor (BDNF), facilitated by PA^20^, thereby preventing the onset of depressive symptoms. Additionally, moderate physical activity has anti-inflammatory effects^21^, which not only reduce the circulating count of leukocytes^22^ but also enhance antioxidant capacity, mitigating oxidative stress^23,24^. These actions contribute to reducing the severity of depressive symptoms.

Reviewing previous studies, we find that immune cells and physical activity can independently affect depressive symptoms. However, which specific immune cells are involved? What is the exact pathway through which physical activity impacts depressive symptoms? In light of this, the current study aims to conduct a statistical analysis based on samples from the NHANES database to observe differences in the counts of various immune cells under total blood cell analysis between populations with depressive symptoms and healthy populations. This study seeks to identify which immune cell has a distinguishing role in depressive symptoms and to explore the moderating effect of physical activity as a variable in the relationship between immune cells and depressive symptoms, evaluating the specific impact. This will provide scientific basis for identifying and improving depressive symptoms in the future.

## Subjects and methods

### Research subjects

This study is based on data from the NHANES database (National Health and Nutrition Examination Survey, NHANES). Managed by the U.S. Centers for Disease Control and Prevention (CDC), the NHANES has been surveying population health since 1999 through questionnaires and physical examinations. The questionnaire covers demographics, socio-economics, diet, and health information, while the physical examination includes physiological measurements and checks. The aim is to assess the health and nutritional status of adults and children (https://wwwn.cdc.gov/nchs/nhanes). All research has been approved by the National Center for Health Statistics (NCHS) Institutional Review Board, and informed consent has been obtained from all participants.

### Data inclusion and exclusion criteria

**Figure 1:**
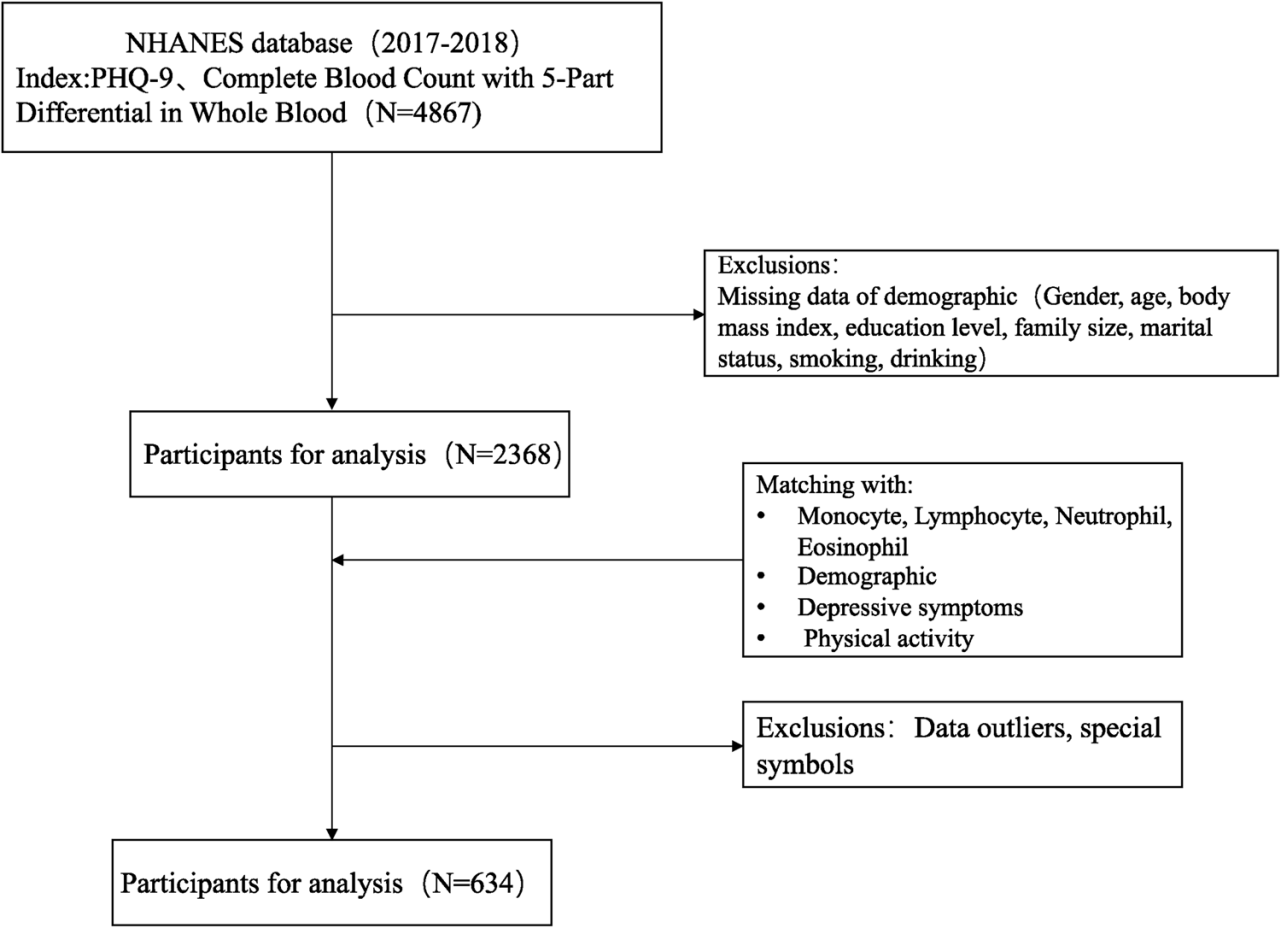
Flow chart for participants recruitment of this study.

This study selected data from the NHANES database for the years 2017–2018 that included demographic information, physical activity, depressive symptoms, and complete blood count. On this basis, a cross-sectional survey was conducted. By clarifying the inclusion and exclusion criteria, a total of 634 participants were ultimately included for analysis (Fig. 1).

### Data collection

#### Demographic information

The study included consolidated demographic information such as gender, age, education level, marital status, number of family members, smoking, drinking, and Body Mass Index (BMI). The BMI is calculated as weight (kg) divided by the square of height (meters).

#### Depressive symptoms

The Patient Health Questionnaire-9 (PHQ-9) is commonly utilized to assess the psychological health status and depressive symptoms of subjects over the past two weeks. Based on the PHQ-9, there are nine questions with options ranging from "Not at all," "Several days," "More than half the days," to "Nearly every day," corresponding to scores from 0 to 3, respectively. The total score for a subject is the sum of all question scores, with a PHQ-9 total score of 0–4 indicating no depressive symptoms; 5–9 indicating mild depressive symptoms; 10–14 indicating moderate depressive symptoms; 15–19 indicating moderately severe depressive symptoms; and 20–27 indicating severe depressive symptoms. The scale has a Cronbach's alpha of 0.839, and the Pearson correlation coefficient between the nine items ranges from 0.160 to 0.578 (*P* < 0.01), demonstrating that the questionnaire has good reliability and validity^25^. In this study, individuals with a score greater than 4 on the PHQ-9 scale were selected as subjects.

#### Immune cell count

The Beckman Coulter DxH 800 instrument is utilized for producing Complete Blood Counts (CBC) on blood samples, examining the blood indices of subjects. CBC is a routine blood test used to assess overall health status and detect conditions including anemia, infections, and leukemia^26^. The sample collection process adheres to Westgard rules^27^, which include submitting progress reports quarterly, calibration of instruments and reagents, and controlling any variable factors to ensure the authenticity and reliability of the data. In this study, the primary data collected from the complete blood cell count include lymphocyte count, basophil count, neutrophil count, and monocyte count, measured in units of 1000 cells/μl.

#### Physical activity

MET (Metabolic Equivalent of Task) refers to the oxygen consumption rate necessary for metabolism at rest. Based on the energy expenditure of quiet, seated activities^28^, different types of physical activities have varying MET values. The NHANES database provides recommended MET values for different types of activities, including vigorous work-related activity (MET = 8), moderate work-related activity (MET = 4), walking or cycling for transport (MET = 4), vigorous leisure-time physical activity (MET = 8), and moderate leisure-time physical activity (MET = 4). The amount of physical activity (PA) in MET-hours per week (MET-h/wk) that an individual engages in at a certain intensity is calculated as MET × frequency per week × duration. The total amount of physical activity (PA) is the sum of all physical activities calculated from their respective MET values^28,29^.

## Statistical analysis

All continuous variables were represented by mean ± standard deviation (Mean ± SD), and categorical variables were denoted by N. The independent samples t-test and chi-square test were employed to calculate the differences in variables between individuals with and without symptoms of depression, aiming to identify "targets"; Z-tests were used for subgroup analyses to compare differences among different populations. Logistic regression analysis was utilized to explore the relationship between immune cells and depression symptom scores, with results expressed in odds ratios (OR) and 95% confidence intervals (95% CI). The PROCESS v4.1 plugin in SPSS 26.0 analysis software was used for moderation effect analysis between total physical activity, neutrophil counts, and depression symptom scores. Standard errors and Bootstrap confidence intervals were obtained by drawing 5000 Bootstrap samples; significance was indicated if the confidence interval did not include 0. The significance level α was set at 0.05. All data in this study were statistically processed and analyzed using SPSS 26.0 and R language version 4.2.2, with *P* < 0.05 indicating a statistically significant difference.

## Results

### Basic demographic characteristics

**Figure 2:**
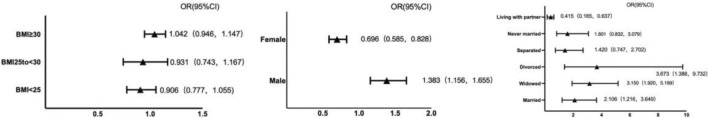
Forest plot.

As illustrated in Table 1, this study ultimately included 634 participants, with 181 individuals identified as having symptoms of depression, resulting in a detection rate of 28.54%. Demographic results revealed significant differences between the depressive symptoms group and the non-depressive symptoms group in terms of gender, BMI index, and marital status (*P* < 0.05), while no significant differences were observed in other variables. Subgroup analysis results (Fig. 2) showed that, after adjusting for confounding factors, the rates of inducing depressive symptoms were higher in males (OR = 1.383; 95%CI 1.156–1.655; *P* < 0.05), those who were divorced (OR = 3.673; 95%CI 1.386–9.732; *P* < 0.05), and individuals with a BMI ≥ 30 (OR = 1.042; 95%CI 0.946–1.147; *P* < 0.05). In immune cell counts, the "target" shows; in the non- depressive symptoms group, the counts of neutrophils and basophils were 4.239 ± 1.7381 and 0.058 ± 0.0507 (1000 cells/µL) respectively, while in the depressive symptoms group, the counts were 4.715 ± 1.8452 and 0.07 ± 0.0505 (1000 cells/µL) respectively, with both differences being statistically significant (*P* < 0.05). There was no statistical significance in the differences in lymphocyte and monocyte counts.

**Table 1:** Basic demographic characteristics.

Variable	Non-depressive symptoms group	Depressive symptoms group	*P*
Age	58.79 ± 16.695	56.06 ± 15.894	0.055
Sex			*P* < 0.05
Male	277	80	
Female	176	101	
BMI (kg/m^2^)	27.39 ± 4.932	28.38 ± 5.468	*P* < 0.05
Education level			0.533
Less than 9th grade	30	13	
9–11th grade	68	33	
High school graduate/GED or equivalent	113	48	
Some college or AA degree	158	63	
College graduate or above	84	24	
Family size	2.77 ± 1.509	2.75 ± 1.606	0.847
Marital status			*P* < 0.01
Married	234	60	
Widowed	58	30	
Divorced	61	48	
Separated	10	9	
Never married	49	17	
Living with partner	41	17	
Smoking			0.071
Yes	158	77	
No	295	104	
Drinking			0.243
Yes	443	174	
No	10	7	
Monocyte (1000 cell/µL)	2.238 ± 1.5916	2.317 ± 0.8245	0.413
Lymphocyte (1000 cell/µL)	0.604 ± 0.185	0.619 ± 0.2525	0.462
Neutrophil (1000 cell/µL)	4.239 ± 1.7381	4.715 ± 1.8452	*P* < 0.01
Eosinophil (1000 cell/µL)	0.058 ± 0.0507	0.07 ± 0.0505	*P* < 0.01

### Exploring the identification of depressive symptoms through immune cell counts

Table 2 presents the relationship between the counts of four types of immune cells and depressive symptoms scores. In Model 1, logistic regression results indicated that only neutrophil counts could significantly predict and identify the risk of depressive symptoms, with an odds ratio (OR) of 1.12 and a 95% confidence interval (CI) of 1.011–1.24. After adjusting for confounding factors such as age, gender, BMI, educational level, family size, marital status, smoking, and drinking, the results of Model 2 demonstrated that neutrophil counts still significantly predicted and identified the risk of depressive symptoms (OR = 1.13, 95% CI 1.02–1.251).

**Table 2:** Immune cells and the risk of depressive symptoms.

Immune cell (1000cell/uL)	Model1 OR [95% confidence intervals]	Model2 OR [95% confidence intervals]
Monocyte	1.02 [0.908, 1.145]	1.015 [0.871, 1.183]
Lymphocyte	0.587 [0.220, 1.568]	1.841 [0.759, 4.468]
Neutrophil	1.12 [1.011, 1.24]*	1.13 [1.02, 1.251]*
Eosinophil	1.137 [0.617, 1.379]	1.155 [0.47, 1.446]

Model 1 Did not adjust for confounding factors.

Model 2 Adjusted for confounding factors including age, gender, BMI, educational level, family size, marital status, smoking, and drinkin*g*.

*P < 0.05.

### The moderating effect of physical activity and neutrophil counts on depressive symptoms

**Figure 3:**
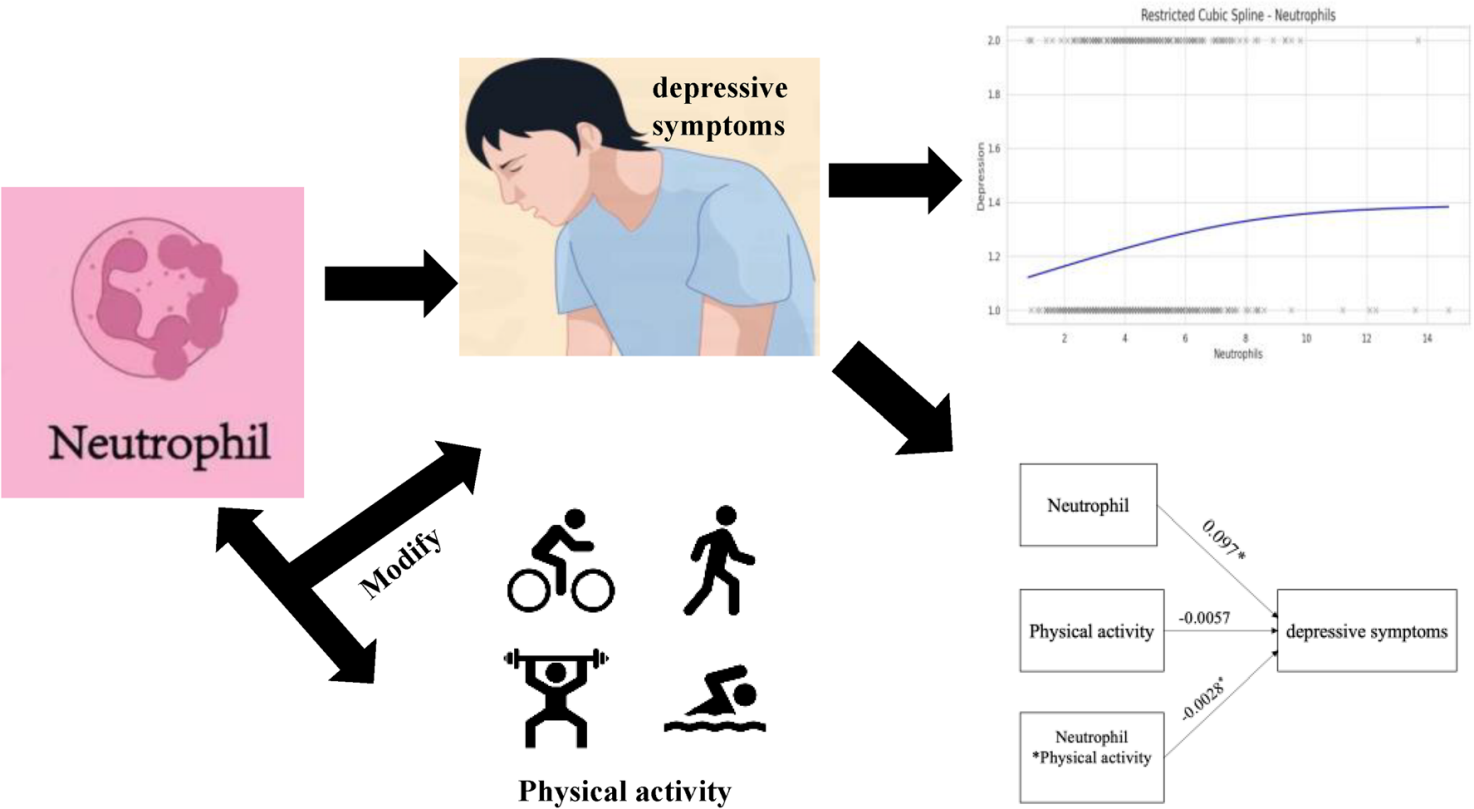
Structural relationship among physical activity, depressive symptoms, and neutrophil count.

To investigate the potential interactive effect between physical activity and neutrophil counts on depressive symptoms, a moderation effect model was developed using the total scores from the PHQ-9. The results (Table 3) revealed an F = 11.0468 with a significance level of *P* < 0.05. This indicates that the null hypothesis (all regression coefficients are equal to zero) can be rejected, suggesting that the moderation effect model is statistically significant. The results (Table 4) highlighted a significant positive correlation between neutrophil counts and depressive symptom levels (coeff = 0.097, *P* < 0.05). This suggests that, when controlling for physical activity, an increase in neutrophil counts is associated with an increase in the severity of depressive symptoms. Conversely, while keeping neutrophil counts constant, there was a negative relationship between the total amount of physical activity and depressive symptoms, though this relationship did not reach statistical significance (coeff = − 0.0057, *P* = 0.0886).Therefore, to elucidate the specific interactions among these three variables, the total amount of physical activity was considered as a moderating variable. The results (Table 4, Fig. 3) indicated that physical activity and neutrophil counts interact in their effects on depressive symptoms, and this moderating effect was statistically significant (coeff = − 0.0028, *P* < 0.01). Specifically, under conditions of physical activity, the positive relationship between neutrophil counts and depressive symptoms was found to be weakened.

**Table 3:** Model summary.

R	R^2^	F	*P*
0.2236	0.05	11.0468	*P* < 0.01

**Table 4:** The moderation effect analysis.

	Coeff	SE	*P*	95%Cl
Neutrophil count	0.097	0.0203	*P* < 0.05	[0.0572, 0.1369]
Physical activity	− 0.0057	0.0034	0.0886	[− 0.0123, − 0.0009]
Neutrophil count * Physical activity	− 0.0028	0.0009	*P* < 0.01	[− 0.0046, − 0.0009]

Dependent Variable: depressive symptoms score.

## Discussion

The previous research has shown that depressive symptoms are associated with increased levels of inflammation in the body^16,30^, primarily due to elevated levels of inflammatory markers such as C-reactive protein (CRP) and interleukin-6 (IL-6) in patients with depression^31^. Based on data from the NHANES database, this study found that monocyte, lymphocyte, and basophil counts could not serve as exposure factors for depressive symptoms. This may be because the relationship between these specific cell types and the inflammatory process of depression is not clear, or their changes are not significant enough to reflect alterations in the immune status associated with depression. Specifically, while these cells play a crucial role in immune responses, their expression in different physiological and pathological states can be influenced by various factors^16,30,32^. For example, the role of monocytes in depression may primarily relate to long-term immune regulation rather than direct association with short-term pathological changes^32^. Lymphocytes, particularly T cells and B cells, are involved in specific immune responses that may be part of prolonged neuroinflammation and autoimmune processes, which are often not directly detectable through routine blood tests^33^. Additionally, basophils, primarily associated with allergic reactions and parasitic infections, have an unclear role in emotional regulation^34^.However, this study found a positive relationship between neutrophil count and depressive symptoms. Neutrophils, a type of white blood cell, serve as primary markers of the body's response to infections^35^, and an excessive increase in their count may reflect intensified inflammation within the body, potentially affecting the neurobiological mechanisms in the brain. Research has confirmed^36^ that neutrophils can activate glial cells in the brain, such as astrocytes and microglia, leading to the release of large amounts of cytokines, such as tumor necrosis factor-alpha(TNF-α)and interleukin-1beta(IL-1β). These cytokines cross the blood–brain barrier, further enhancing the inflammatory response and affecting neurotransmitter synthesis and release, as well as neuron growth and survival, which could potentially trigger depressive symptoms^37–40^. Therefore, from a clinical perspective, although the diagnosis of depressive symptoms relies on the assessment of subjective scales, including neutrophil counts could make the diagnostic results more precise.

This study also found that there is no direct correlation between physical activity (PA) and depressive symptoms. A possible explanation is that depressive symptoms are triggered by a combination of genetic, environmental, and psychosocial factors. While physical activity is an important factor influencing mental health, its effects may be obscured by other exposure factors such as life event stresses and lack of social support^41,42^. However, the study discovered that when combined with neutrophil count, physical activity (PA) has a significant moderating effect, effectively mitigating the impact of neutrophil count on depressive symptoms. The possible reason is that physical activity influences immune function by altering the number and activity of neutrophils. When the body is in an "anti-inflammatory" state, physical activity increases the production of anti-inflammatory cytokines such as IL-4 and IL-10, inhibiting the activation of neutrophils and the production of inflammatory factors^43,44^.Furthermore, from the perspective of neurotransmitters and hormones, physical activity can regulate levels of serotonin and dopamine in the brain^45^. These neurotransmitters are thought to help regulate mood and suppress inflammation^46^, while also having analgesic and pleasure-inducing effects that positively impact emotions. Additionally, physical activity increases the secretion of hormones such as cortisol and growth hormones, which have inhibitory effects on the activation of neutrophils and the production of inflammatory factors. From an antioxidative mechanism perspective, physical activity enhances the body's antioxidant capacity, such as increasing the activity of superoxide dismutase and catalase^47^. This helps neutralize free radicals, thereby reducing cell damage and inflammation. Regarding neuroplasticity, physical activity effectively increases neuroplasticity^48–50^ by raising levels of neurotrophic factors such as brain-derived neurotrophic factor (BDNF), thereby promoting neuron growth and survival, as well as the formation and repair of synapses. Therefore, when engaging in physical activity, the body can inhibit the activation of neutrophils and the production of inflammatory factors through multiple biological mechanisms, thereby reducing the risk of developing depression.

This study also found significant differences between the depressive symptoms group and the non-depressive symptoms group regarding gender, BMI, and marital status. Subgroup analysis revealed that males, those who are divorced, and individuals with a BMI ≥ 30 seem to be more sensitive to depressive symptoms. Firstly, existing research has already shown that gender is a significant factor in triggering depression, although most studies indicate that women have a higher incidence rate of depression than men^51,52^. This may suggest that in certain environmental exposures or cultural contexts, men may be less likely to seek help or express emotions when faced with life and work pressures, thereby increasing their risk of depressive symptoms^53^.Secondly, a BMI ≥ 30, indicative of obesity, has been shown to be correlated with depressive symptoms. The relationship between obesity and depression may be bidirectional: obesity can increase the risk of depression and vice versa^54,55^. Research has also demonstrated that obesity might increase the risk of depressive symptoms through biological mechanisms such as inflammation and hormonal imbalances, as well as psychosocial mechanisms such as dissatisfaction with body image and social discrimination^56^. Lastly, divorce has been found to be associated with a higher risk of depression. Changes in marital status often accompany significant psychological stress and changes in lifestyle, which could have a long-term negative impact on an individual’s mental health. Additionally, divorce could lead to reduced social support, thus triggering depressive symptoms^57,58^.These findings suggest that future researchers and mental health professionals should focus on the prevention and treatment of depressive symptoms in special populations and advocate for the adoption of healthy lifestyles.

## Conclusions and suggestions

This study suggests that a significant increase in neutrophil count may serve as a potential contributing factor to the manifestation of depressive symptoms; physical activity may mitigate the impact of neutrophil count on depressive symptoms. Therefore, future directions include: 1. Enhanced monitoring of neutrophils and other immune cells in diagnosing individuals with depressive symptoms; 2. The recommendation for various societal groups to engage in physical activity to a certain extent. This aims to promote mental health and prevent the onset of psychological disorders.

## Limitations and future directions

1. The data for this study were exclusively derived from the NHANES database for 2017–2018, making it impossible to ascertain if the subjects had impaired cognitive functions during data collection. Future research should refine the inclusion and exclusion criteria of subjects to enhance the reliability of the results.
2. The study is primarily based on a cross-sectional analysis utilizing the NHANES database, thereby limiting the ability to infer causal relationships between variables. Further research with a larger sample size is needed to explore the specific relationships between physical activity, neutrophil count, and depressive symptoms. This includes analyzing the predictive power of neutrophil count in identifying depression and incorporating the five essential elements of physical activity into longitudinal experiments to develop precise interventions for the prevention and amelioration of depressive symptoms.
3. The assessment of physical activity was subject to substantial human bias due to the subjective nature of the questionnaire used, and the GPAQ's scoring standards did not clearly differentiate between the three intensities (low, moderate, high) of physical activity. Consequently, the interrelations between different intensities of physical activity, neutrophil count, and depressive symptom scores were not dissected. Future studies should attempt to use objective instruments for measuring physical activity to increase the accuracy of the research.
4. The PHQ-9 is a self-reported questionnaire for depressive symptoms, not a clinical diagnosis, which may introduce bias. Future studies should aim to use objective tools for assessing depressive symptoms to enhance the accuracy of the research.

## Data Availability

All NHANES data for this study are publicly available and can be found here: https://wwwn.cdc.gov/nchs/nhanes. The datasets used and/or analysed during the current study available from the corresponding author on reasonable request.
